# Supplementary material for: The Impact of the COVID-19 Pandemic on Rabies Reemergence in Latin America: the case of Arequipa, Peru
Source: medRxiv. 2020 Aug 13:2020.08.06.20169581. Preprint. [Version 2] doi: 10.1101/2020.08.06.20169581 (PMC7430610; doi:10.1101/2020.08.06.20169581)
Supplement: Supplement 2020 [file 92571-2020.08.06.20169581-1.docx]

**Supplementary Table 1**

| **Year** | **Coverage estimate** |
| --- | --- |
| 2014 | 48.98% |
| 2015 | *48.98% |
| 2016 | 61.48% |
| 2017 | 49.90% |
| 2018 | 52.85% |
| 2019 | 58.5% |

*Due to lack of reliable data, we relied on the estimate from 2014 for 2015

**
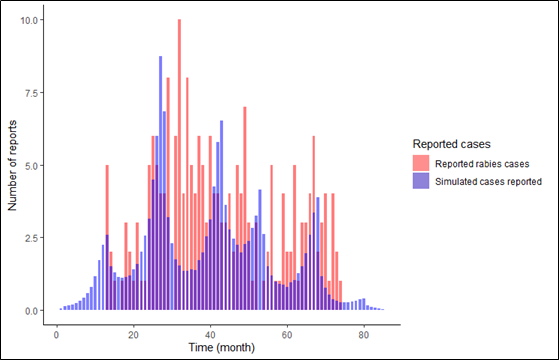
**

**Supplementary Figure 1**

Reported rabies case data are depicted in pink bars and rabies cases simulated by the model are depicted in blue. The model is parameterized by fitting monthly incidence assuming a 10% reporting rate.


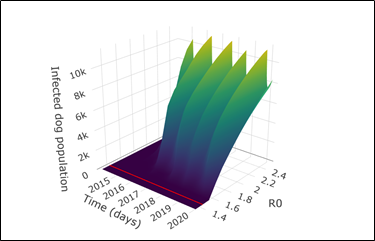


**Supplementary Figure 2**

Figure S2 depicts the simulated number of infected dogs for a wide range of R0. We show all possible values from 1.36, the low estimate found from focus control data to 2.0, a high end estimate from the rabies literature. The red line shows the transect R0=1.44 which is our best fit estimate for Arequipa. Though the scale makes the line look flat in this image, in Figure 2C in the main text the regional dynamics are displayed at a narrower range.
